# Active and avoidant coping profiles in children and their relationship with anxiety and depression during the COVID-19 pandemic

**DOI:** 10.1038/s41598-022-15793-4

**Published:** 2022-08-04

**Authors:** Qiaochu Zhang, Yanlin Zhou, Samuel M. Y. Ho

**Affiliations:** 1 Department of Social and Behavioural Sciences, College of Humanities and Social Sciences, Floor 7, AC1, City University of Hong Kong, Tat Chee Avenue, Kowloon, Hong Kong; 2 Department of Psychology and Behavioral Sciences, Zhejiang University, 148 Tianmushan Rd, Xixi Campus, Hangzhou, China

**Keywords:** Psychology, Human behaviour

## Abstract

Active and avoidant coping styles are important dispositional factors in the development of anxiety and depression symptoms. Children use both active and avoidant coping strategies together in daily life. No studies have investigated the relationship between active–avoidant coping profiles and internalizing symptoms in children. The present study aimed to investigate children’s active–avoidant coping profiles and assess the relationship that active–avoidant coping profiles have with anxiety and depression symptoms. A two-wave longitudinal study was conducted among 322 Chinese children in the People’s Republic of China during the COVID-19 pandemic. Participants completed the Children’s Coping Strategies Checklist-Revised 1 at Time 1 and the Revised Child Anxiety and Depression Scale at Time 1 and 6 months later (Time 2). Four active–avoidant coping profiles were revealed: low active copers, high active copers, balanced copers, and avoidant copers. Low and high active copers had lower levels of anxiety and depression symptoms than balanced copers and avoidant copers. Avoidant copers showed a larger decrease in depression symptoms than balanced copers and high active copers during the COVID-19 pandemic. It is important to improve children’s active–avoidant coping profiles to relieve anxiety and depression symptoms.

## Introduction

Dispositional coping styles refer to a trait-like tendency to respond to stressors using a certain type of strategies across situations^1,2^. Children’s dispositional coping styles affect their ability to manage stress^3^. Amid the COVID-19 pandemic, children and adolescents have experienced increased stress due to infection control measures, such as lockdowns and vaccinations^4,5^. Many of them may feel threatened by the increased risk of infection.

Coping behaviour was reported to provide a buffer against the negative impact of COVID-19-related stressors on mental health^6^. For instance, Mi et al.^6^ used a self-developed scale to measure coping and reported that more coping behaviour may reduce the negative effect of COVID-19-related stressors on depression and anxiety symptoms. However, the authors did not measure active coping and avoidance coping separately. Previous cross-sectional studies have revealed that active–avoidant coping styles are related to anxiety and depression during the COVID-19 pandemic in adults^7–11^. The distinction between active and avoidant coping styles is also important in the literature on children’s coping styles during the COVID-19 pandemic^12–14^.

Most studies focus on the relationship of an individual coping style to anxiety and depression[e.g.,^13^]. People rarely rely on a single coping style; instead, they have coping profiles consisting of multiple coping styles[e.g.,^15^]. However, no research has investigated children’s active and avoidant coping profiles. Therefore, it is unclear whether children with more differences between active and avoidant coping styles show more or less internalizing symptoms than children with an equal level of active and avoidant coping styles. Without improved knowledge of children’s active–avoidant coping profiles, psychologists lack evidence-based judgement about how to improve children’s active–avoidant coping patterns in the context of the COVID-19 pandemic.

### Coping profiles in young people

Latent profile analysis can reveal heterogeneous coping profiles in children; it examines and compares different models for researchers to find the best method to categorize heterogeneous children into homogeneous groups of coping profiles comprising multiple dispositional coping styles^16^.

Using latent profile analysis, Herres^17^ identified four distinct coping profiles for adolescents with a mean age of 16 years: disengaged copers (16.1%), independent copers (39.5), support-seeking copers (31.6%), and active copers (12.8%). Disengaged copers had the lowest scores on the overall coping scales and used avoidant coping strategies more than others. Independent copers used a moderate level of coping strategies with a relatively lower support-seeking strategy. Support-seeking copers reported a relatively higher level of support-seeking strategy than other strategies. Active copers reported the highest amount of all coping strategies. Contrary to the findings that the avoidant coping style was maladaptive, the study revealed that support-seeking copers and active copers showed a higher level of separation anxiety, generalized anxiety, and total anxiety than independent and disengaged copers. Using latent profile analysis, Perzow, Bray^16^ also identified four coping profiles of adolescents (12–18 years) in both a community sample and a low-SES sample: inactive, cognitive, and engaged copers and active copers. Engaged copers (21% in the community sample and 16% in the low-SES sample) used more engagement strategies and fewer disengagement strategies, and they showed the lowest level of anxiety and depression. Active copers (10% in the community sample and 13% in the low-SES sample) who had above-average scores in all coping styles had the highest level of depression and anxiety symptoms. During the COVID-19 pandemic, a study revealed three coping profiles in adults to cope with stressors: the engaged profile (57.6%), which consisted of active coping, planning, acceptance, and positive reframing; the disengaged profile (26.9%), which comprised low problem-focused coping, low social support, low acceptance, and low positive reframing; and the avoidant profile (15.4%), involving substance use, self-blame, and humour. Adult participants with the engaged profile had the highest well-being, and those with the disengaged profile had the highest anxiety. Adults with the avoidant profile had the poorest well-being^7^. Although these studies revealed coping profiles in adults and examined their relationship with anxiety or depression, none of them were longitudinal. Thus, they failed to examine how coping profiles affect anxiety or depression during the COVID-19 pandemic in the long term.

Little research has investigated coping profiles in children during the COVID-19 pandemic. Although the existing studies illustrated the importance of adolescents’ and children’s coping profiles as correlates of anxiety and depression symptoms, they cannot illustrate the relationship between active–avoidant coping profiles and internalizing symptoms in children. Additionally, longitudinal studies on coping profiles are scarce. Longitudinal design can test how coping profiles affect changes in internalizing symptoms over time^18^. Therefore, the current study adopted a longitudinal design and focused on children’s active–avoidant coping profiles. We first examined whether children’s coping profiles could be categorized based on the difference between active and avoidant coping styles. Then, we tested how active–avoidant coping profiles contributed to anxiety and depression symptoms over time during the COVID-19 pandemic.

### Avoidance versus active dispositional coping styles

Maladaptive avoidance is often related to adverse emotional outcomes^19^. Child avoiders avoid stressors and use coping strategies, including repression, disengaging behaviour, and wishful thoughts, to deal with threats^12^. Despite evidence suggesting that avoidant coping can be beneficial in the short term^20^, the majority of coping literature has demonstrated that it is a risk factor for anxiety and depression in the long term. Among a sample of young people from 16 to 24 years, structural equation modelling indicated that higher avoidant coping was associated with more suicidal ideations^21^. Another study recruited 164 adolescents to measure their coping with recurrent pain, anxiety/depression symptoms, and somatic complaints by questionnaires^22^. The study found that avoidant coping was related to higher anxiety and depression levels. Research on coping styles in children with cancer measured coping styles from multiple informants; disengagement coping was associated with a higher level of anxiety and depression reported by multiple formants^23^.

Active coping is a protective factor against internalizing symptoms. The active copers approach stressors by exerting control over the situation; they attempt to solve the problems, make decisions, understand the problem, and think positively in stressful situations^12^.

Overall, active coping is the opposite of avoidant coping in terms of its effect on psychological well-being. For example, in a sample of 870 adolescents, avoidant coping was related to less well-being and greater stress, while active coping was related to higher well-being and less stress^24^. Similarly, a study recruited 168 Latino youth, and the results showed that active coping was related to fewer internalizing symptoms and less posttraumatic stress, while avoidant coping was associated with more internalizing symptoms and more posttraumatic stress^25^. Among adolescents of Mexican origin, a study found that active coping buffered the relationship between stress and internalizing symptoms^26^.

During the COVID-19 pandemic, active and avoidant coping styles have been reported to show opposite effects on psychological wellbeing. Research has suggested that problem-focused coping protects front-line health care workers’ emotional wellbeing in the hospital for inpatients affected by the COVID-19 virus^8^. However, more frequent use of avoidant coping styles has been shown to be associated with higher psychological stress during the pandemic in pregnant women^9,27^. In adults, higher use of an active coping style and lower use of an avoidant coping style were associated with higher anxiety symptoms^10^. Worldwide research involving samples from 12 countries also showed that disengagement from problems was associated with negative mental health outcomes, while problem-focused coping was related to better mental wellbeing^11^. However, another study among pregnant women in Ireland during the COVID-19 pandemic showed that avoidance coping, but not problem-focused and emotion-focused coping, had a significant relationship with psychological distress. More avoidance coping was related to higher psychological distress^28^.

In children from 3 to 12 years old, a study showed that disengagement coping was related to more emotional difficulties, whereas engagement coping was associated with fewer emotional problems during the COVID-19 pandemic^29^. Inconsistent findings were obtained from a study involving students in high school. Research showed that adolescents with disengagement coping had lower distress and COVID-19-related worries, whereas active coping had no association with distress or worries^14^. These studies were cross-sectional and thus failed to investigate how active and avoidant coping may affect the changes in anxiety and depression symptoms in the long term. A longitudinal study showed that 1 year after the beginning of the COVID-19 pandemic, an active coping style predicted better mental health, while nonactive coping predicted worse mental health^13^. Despite evidence clearly showing the importance of active or avoidant coping styles in mental health during the COVID-19 pandemic, these studies investigated active or avoidant coping styles separately and failed to reveal active–avoidant coping profiles in children.

Active and avoidant coping reflects whether children orient towards or away from threats^20^. However, people are not either avoiders or active copers exclusively. An experiment found that a group of adult participants had high scores on both avoidant and approach coping styles above medians, showing avoidant-approach coping conflict after receiving both avoidant and approach coping training^30^. Avoidant-approach coping conflict was related to higher posttraumatic stress symptoms. Aldridge and Roesch^31^ examined coping typologies among a sample of adolescents from minority socioeconomic families with latent profile analysis. The study identified three groups by using the 60-item COPE scale^32^: low generic copers (44.4%), active copers (48.3%), and avoidant copers (7.3%). Low generic copers showed a low level of all coping strategies. Active copers relied more on active coping strategies than avoidant coping strategies, while avoidant copers adopted more avoidant coping than active coping. Therefore, some people have a high or low coping profile on both active and avoidant coping, whereas others may be lower in either active or avoidant coping styles. Additionally, the individual difference in active–avoidant coping patterns is likely to be consistent over time^20,33^. Thus, the active–avoidant coping profile may be dispositional. However, no study has investigated children's dispositional active-–avoidant coping profiles, limiting our understanding of how active–avoidant coping patterns are related to internalizing symptoms during the COVID-19 pandemic. For example, it is unknown whether coping patterns that are equally high on both active and avoidant coping are associated with more or less anxiety and depression than the high avoidant coping pattern.

### The current study

The current study investigated children’s active–avoidant coping profiles and their relationship to anxiety and depression symptoms in the context of the COVID-19 pandemic. Children were involved in a two-wave longitudinal study during the COVID-19 pandemic over 6 months. The first assessment was conducted from November 16 to November 30, 2020 (Time 1). Although vaccination had started in mainland China, infection cases of COVID-19 were reported on a daily basis in mainland China during the first assessment. Additionally, primary schools in Shenzhen, a city in mainland China, strictly implemented preventive measures, including social distancing. Research has reported that social distancing is related to increased mental health concerns^34^. During the assessment, children reported how they coped with stressors related to the COVID-19 pandemic, as well as their anxiety and depression levels. After approximately 6 months, the second assessment started from May 27 to June 9, 2021 (Time 2). Children reported their level of anxiety and depression in the second assessment. During the second assessment, the risk of COVID-19 infection increased in early June as more cities in Guangdong were categorized into medium- to high-risk areas, and the number of newly diagnosed cases reported each day slightly increased. The quarantine measures were implemented more strictly; an increased number of people needed to be in quarantine for at least 14 days. Mental health was likely to worsen during a lengthy quarantine period^35^.

The current study aimed (1) to examine the relationship of active–avoidant coping profiles to the overall level of anxiety and depression symptoms across 6 months, and (2) to investigate how different active–avoidant coping profiles interacted with changes in anxiety and depression symptoms. It should be noted that data collection was conducted during the COVID-19 pandemic. The results reflected participants’ coping with the additional stressors related to COVID-19 mentioned above.

### Hypotheses

Previous research has shown that, to cope with stressful situations, such as the COVID-19 pandemic, adults have three coping profiles: engaged profiles, disengaged profiles and avoidant profiles^7^. It was hypothesized that similar coping profiles would also be revealed in children. Consistent with a previous study on active–avoidant coping styles in children during the COVID-19 pandemic^29^, an active coping style may better protect children from anxiety and depression symptoms than an avoidant coping style in the face of an increased risk of COVID-19 infection and stressful preventive measures during the period of the present study. Thus, it was hypothesized that active copers would show the lowest level of anxiety and depression symptoms, and avoidant copers would show the highest level of anxiety and depression symptoms. Because from Time 1 to Time 2, preventive measures and the risk of COVID-19 infection increased, the study hypothesized that the interaction between coping profiles and time would be significant; children with an active profile would show the smallest increase in anxiety and depression levels, while those with an avoidant profile would demonstrate the largest increase in anxiety and depression levels.

## Methods

### Participants

Data were obtained from a large longitudinal research project in which 321 Chinese children were recruited from a primary school in Shenzhen, Guangdong Province, China. The primary school was located in a community of the middle class. These children were aged from 9 to 11 years (mean = 9.54; *SD* = 0.51), with 53.6% (*n* = 172) girls, 45.5% (*n* = 146) boys, and three not indicating their gender. The majority of children (91.6%) were born in mainland China (*n* = 294); 8.4% (*n* = 27) of children were born in Hong Kong. Among the 321 children who completed the first assessment, 304 (*n*_boys_ = 142; *n*_girls_ = 159) continued to conduct the second assessment. The attrition rate was approximately 5.6%. The attrition resulted from the participants being absent from the school for personal reasons (e.g., transferring to other schools) during the second assessment.

### Measurement

#### Avoidant and active coping styles

Avoidant and active coping styles were measured by the Children’s Coping Strategies Checklist-Revised 1 (CCSC-R1)^36^. A Chinese version of the scale was developed by standard translation and back-translation procedures^37^. The confirmatory factor analysis showed a 21-item active coping scale and a 5-item avoidant coping scale (refer to Table S.1 in the Supplementary Information). Items were rated on a four-point Likert scale (1 = *never*, 4 = *most of the time*; “when you encountered stressors in the past month, you tried to ignore it”). An avoidant coping score and an active coping score were calculated by averaging scores for each item. The higher the scores are, the stronger the disposition to use avoidant or active coping. The Chinese translation of the scale showed good internal consistency in the study (avoidant coping: Cronbach’s alpha = 0.74; active coping: Cronbach’s alpha = 0.94).

#### Anxiety and depression symptoms

The Chinese version of the Revised Child Anxiety and Depression Scale^38^ was downloaded from the website (http://www.childfirst.ucla.edu/Resources.html). The scale included 47 items. The Anxiety Disorders subscale has 37 items (e.g., “afraid of looking foolish in front of people”), and the Major Depressive Disorder subscale includes 10 items (MDD; “feels nothing is much fun anymore”). Items were rated on a Likert scale from 0 to 3 (0 = *never*; 1 = *sometimes*; 2 = *often*; 3 = *always*). An anxiety score and a depression score were calculated by summing the scores of the relevant items on the Anxiety Disorders subscale and Major Depression subscale, respectively. The scales had excellent reliability with the current sample (Anxiety Disorder scale: Cronbach’s alpha = 0.958; Major Depression Disorder scale: Cronbach’s alpha = 0.878).

#### Personal information

We collected participants’ demographics involving age, gender, and birthplace.

### Procedure

All research procedures were performed in accordance with relevant guidelines and regulations of the Human Subjects Ethics Sub-Committee. Informed consent forms and assent forms were distributed to students in primary school. Students were informed about the purpose and the procedure of the study, and then they provided informed consent to their parents. Parents and/or legal guardians who agreed their children to participate in the survey signed and submitted the informed consent form to researchers.

Only students who signed the informed consent form and obtained it from their parents and/or legal guardians participated in the study. From November 16 to November 30, 2020 (Time 1), participants completed a set of questionnaires that included RCADS and the translated CCSC-R1 in the classrooms. After approximately 6 months (Time 2) from the first assessment, participants completed the second assessment to assess their anxiety and depression symptoms using the RCADS from May 27 to June 9, 2021. Students had approximately 45 min to complete all the questionnaires for both the first and second assessments. A researcher and a teacher were present in all assessment sessions to answer the participants’ questions.

### Statistical analyses

The construct validity of the Chinese version of the active and avoidant coping scale was examined by confirmatory factor analysis (refer to the Supplementary Information). Descriptive statistics were subsequently present. Gender differences in the psychological variables were investigated by independent t tests. The association between age and psychological variables was also tested. Then, two-tailed Pearson’s correlation was conducted to assess the relationship among the psychological variables. Next, we conducted latent profile analysis to identify heterogeneous active and avoidant coping profiles in children at T1 using maximum likelihood estimation with robust standard error (MLR). We increased the number of groups for the model until the fit indices showed that the model was not significantly better than the model with one fewer group. The Bayesian information criterion (BIC) and the Akaike information criteria (AIC) were used to estimate the model fit, with smaller numbers indicating better model fit^39^. Additionally, the value of entropy assesses how well a model classifies individuals; a larger value indicates better model fit^39^. The significant *p* value of the Vuong–Lo–Mendell–Rubin likelihood ratio test (VLMR), the adjusted Lo–Mendell–Rubin likelihood ratio test (Adj. LMR), and the Bootstrapped Likelihood Ratio Test (BLRT) suggested that a model with a certain number of groups was significantly better than the model with one fewer group. Next, chi-square analyses were conducted to assess whether the four groups with distinct coping profiles differed in gender, age, and birthplace. Finally, repeated multivariate analysis of variance (MANOVA) was performed to examine the main effect of time and coping profiles on anxiety and depression levels, as well as the interaction between coping profiles and time over the period of 6 months.

### Ethics approval and consent to participate

Ethics approval was obtained from the Human Subjects Ethics Sub-Committee of the City University of Hong Kong (2020-21-CIR2-B1). Students’ parents who agreed their children to participate had signed the informed consent sheets, and students had signed the assent sheets to participate.

## Results

### Descriptive statistics

At T1, girls were not significantly different from boys in anxiety and depression levels. Additionally, girls were not significantly different from boys in avoidant coping, *t* (316) = 0.029, *p* = 0.977, and active coping, *t* (316) = − 1.07, *p* = 0.285 (refer to Table 1). Pearson’s correlation showed that age was not significantly associated with active coping,* r* = 0.016, *p* = 0.779, avoidant coping, *r* = − 0.049, *p* = 0.390, anxiety symptoms, *r* = − 0.004, *p* = 0.948, or depression symptoms, *r* = − 0.029, *p* = 0.608 at T1. T tests showed that birthplace was not a significant factor affecting active coping, *t* (319) = − 0.514, *p* = 0.608, avoidant coping, *t* (319) = − 0.007, *p* = 0.994, anxiety, *t* (319) = 0.276, *p* = 0.783, and depression, *t* (319) = − 0.270, *p* = 0.787.

**Table 1:** Mean (standard deviation) and correlations of psychological variables (n = 321).

	Boys		Girls		Correlations					
	Mean	(SD)	Mean	(SD)	*r*	*r*	*r*	*r*	*r*	*r*
					T1 active	T1 avoid	T1 anxiety	T1 depression	T2 anxiety	T2 depression
T1 active	54.29	15.06	52.72	13.56	–					
T1 avoidant	7.93	3.24	7.95	2.78	0.046	–				
T1 anxiety	24.01	20.93	28.57	22.60	− 0.076	0.388**	–			
T1 depression	4.95	5.70	5.26	5.54	− 0.186**	0.449**	0.849**	–		
T2 anxiety	11.32	14.23	13.06	15.29	− 0.068	0.361**	0.636**-	0.544**	–	
T2 depression	2.06	3.83	2.16	3.68	− 0.121*	0.329**	0.478**	0.511**	0.837**	–

Active = active coping style. Avoidant = avoidant coping style. **p < .001, *p < .05.

As shown in Table 1, two-tailed Pearson’s correlation was used to examine the relationship of T1 active and avoidant coping with anxiety and depression at T1 and T2. A higher active coping style was significantly related to lower depression symptoms at T1 and T2 (*r* = − 0.186 to − 0.161, *p*s < 0.05), while its associations with anxiety symptoms at T1 and T2 were not significant (*r* = − 0.076 to − 0.068, *p*s > 0.05). Higher avoidant coping was significantly positively associated with anxiety and depression symptoms at T1 and T2 (*r* = 0.329–0.449, *p*s < 0.01).

### Active–avoidant coping profiles in T1

We examined the model fit indices for models with one to five groups (refer to Table S.2 of the Supplementary Information). The four-group model has the smallest BIC and AIC, as well as the highest entropy. All *p* values of VLMR, Adj. LMR and BLRT were insignificant for the five-group model, which suggested that the model was not significantly better than the four-group model. Thus, the four-group model showed the best model fit.

**Figure 1:**
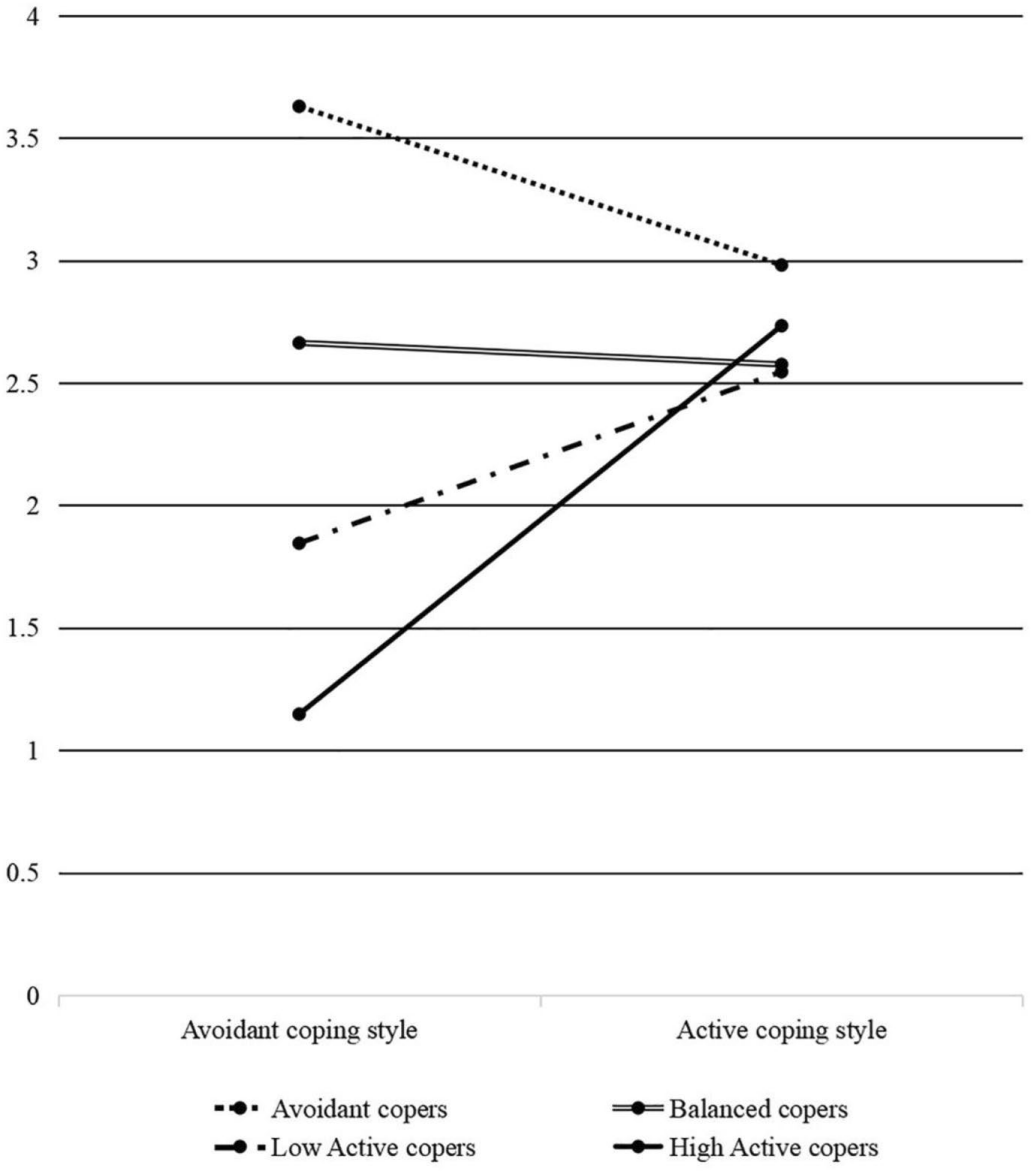
Means scores of active and avoidant coping styles in the four coping profiles.

Avoidant coping was lower than active coping in group one and group three. The difference between active and avoidant coping was larger in group three than in group one. Moreover, an independent t test showed that group three was significantly larger in active coping style than group one, *t* (282) = − 2.61, *p* < 0.05. Therefore, group one was labelled low active copers, and group three was high active copers. The difference between avoidant and active coping was the lowest among the four groups for group two. Thus, group two was termed balanced copers group. Avoidant coping was higher than active coping in group four. Group four was accordingly interpreted as avoidant copers. Low active copers accounted for 35.8% of the sample; balanced copers comprised 9.3%. Approximately half of the participants belonged to high active copers (52.6%). Only a small minority were classified as avoidant copers (2.2%). Figure 1 shows the means of active and avoidant coping styles of the four groups. It should be noted that the four profiles had more variations in the avoidant coping style scores than in the active coping style scores. The four groups were not significantly different in terms of gender, *χ*^*2*^(3) = 6.839,* p* > 0.05, age, *χ*^*2*^(6) = 10.537, *p* > 0.05, and birthplace, *χ*^*2*^(3) = 2.709, *p* > 0.05.

### Anxiety and depression symptoms during the COVID-19 pandemic based on coping profiles

**Figure 2:**
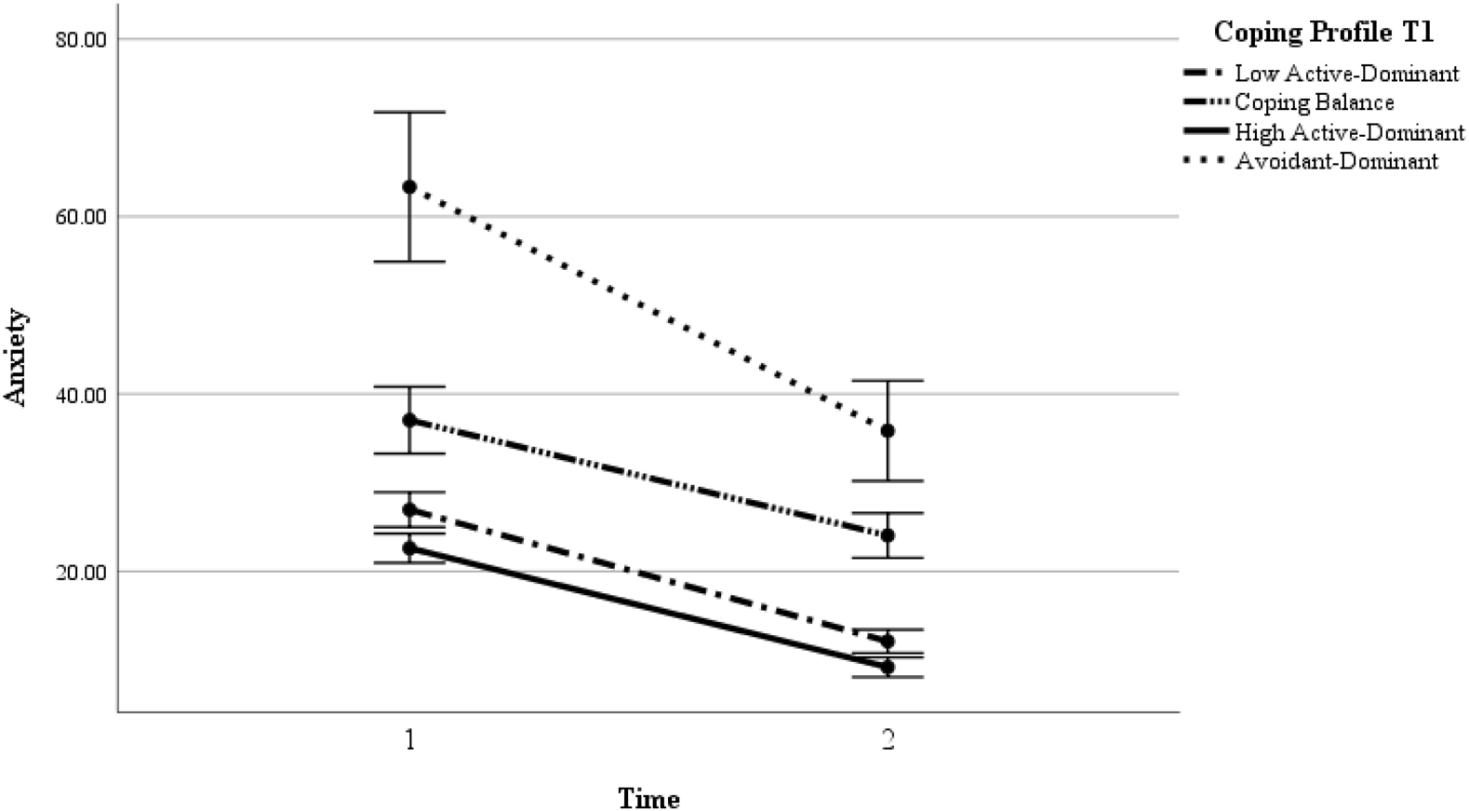
Anxiety across the two times and as a function of coping profiles.

With anxiety symptoms as the outcome variable, repeated MANOVA revealed significant main effects of group and time; however, the interaction between group and time was insignificant (refer to Fig. 2; Table S.3 of the Supplementary Information). The Scheffe post hoc analyses showed that low and high active copers were not significantly different in anxiety symptoms. Low active copers had significantly lower anxiety symptoms than balanced copers and avoidant copers. High active copers had significantly lower anxiety symptoms than balanced copers and avoidant copers. Balanced copers had significantly lower anxiety symptoms than avoidant copers. Overall, anxiety symptoms significantly decreased over the 6 months during the COVID-19 pandemic.

**Figure 3:**
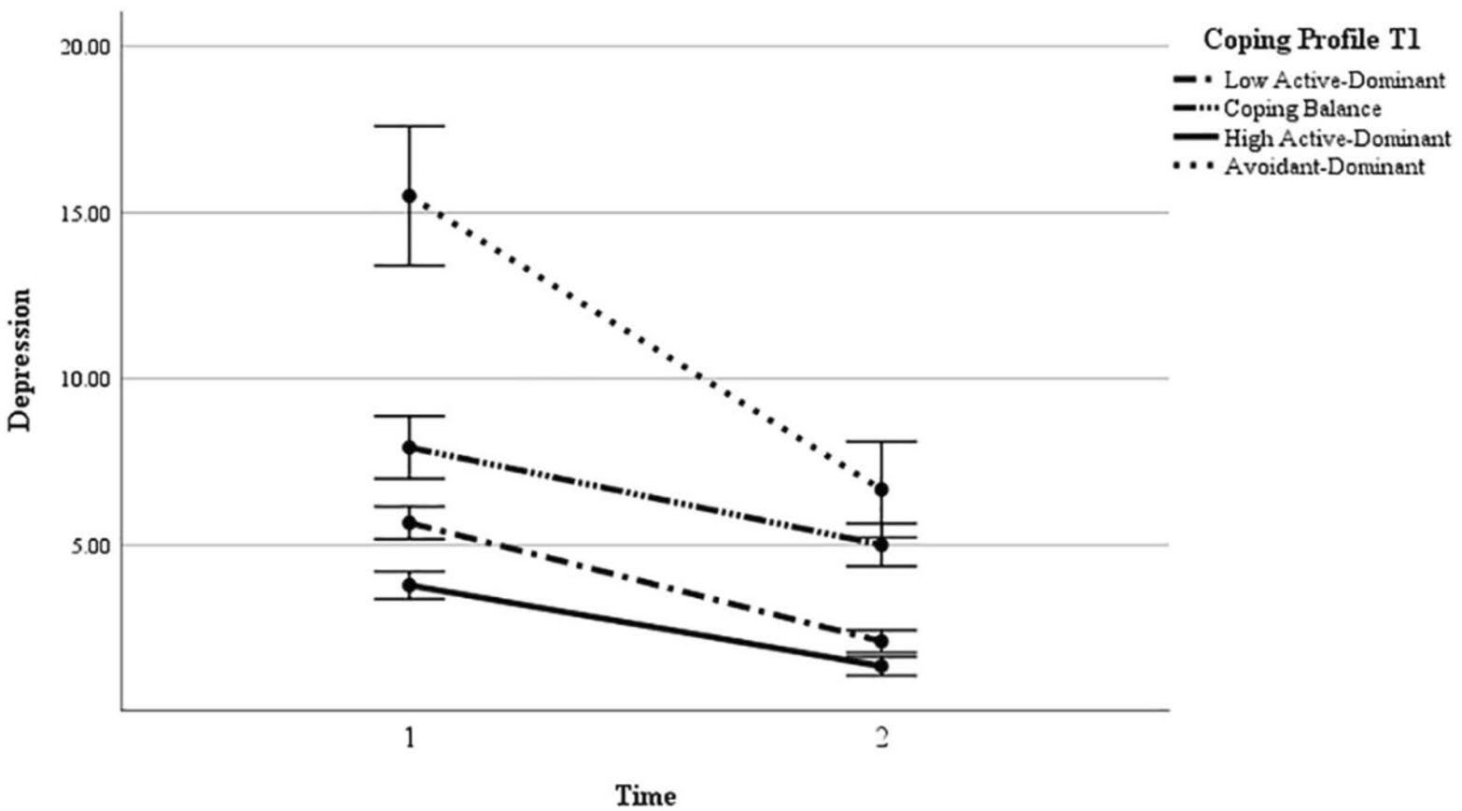
Depression across the two times and as a function of coping profiles.

Repeated MANOVA for depression symptoms revealed a significant main effect of time and group (refer to Fig. 3; Table S3 of the Supplementary Information). The interaction between time and group was also significant. Low active copers had significantly higher depression symptoms than high active copers. Low active copers showed significantly lower depression symptoms than balanced copers and avoidant copers. Balanced copers had significantly lower depression symptoms than avoidant copers and had significantly higher depression symptoms than low active copers and high active copers. High active copers had the lowest depression symptoms, while avoidant copers showed the highest depression symptoms. Overall, depression symptoms significantly decreased from T1 to T2. Subsequently, a one-way ANOVA revealed that the decrease was significantly less for balanced and high active copers than for avoidant copers, *F* (3, 298) = 6.22, *p* < 0.001. The decrease was not significantly different from other comparisons among groups*.*

These results demonstrated that low active copers had the most positive emotional outcomes, while avoidant copers had the most negative emotional outcomes after experiencing stressful situations, including social distancing and an increased risk of infection and quarantine. Balanced copers suffered more from anxiety and depression symptoms than low and high active copers in response to COVID-19-related stressors. Avoidant copers showed a decrease in depression that was significantly larger than balanced copers and high active copers during the COVID-19 pandemic.

## Discussion

The present study aimed to identify active–avoidant coping profiles in children and investigate the association of these profiles with internalizing symptoms over time during the COVID-19 pandemic. With latent profile analysis, the current study revealed a four-group model of active–avoidant coping profiles: low active copers, high active copers, balanced copers, and avoidant copers. We did not find a significant difference between avoidant and active coping profiles by gender, age, or birthplace. Contrary to previous studies suggesting that females showed a higher level of avoidant coping than males^40^, girls were not more likely to be categorized as avoidant copers than boys. Additionally, the study by Aldridge^31^ found that active copers are marginally older than individuals in other groups, which was not replicated in our sample of children.

Our results suggested that nonclinical primary school children adopted both active and avoidant coping styles simultaneously, although the difference between the level of avoidant and active coping styles varied across individuals. The majority (88.8%) of primary school children showed a higher level of active coping style than avoidant coping style. Among them, 53% of children had a high active coper profile, which had a relatively large difference between active and avoidant coping styles. A small group of children (11.3%) showed aberrant coping patterns distinct from those of the majority of the participants. Among them, 9.3% of participants had a similarly high level of both avoidant and active coping styles. In contrast, 2% had a higher avoidant coping style than an active coping style. High and low active copers, as well as avoidant coper profiles in children revealed by the present study, were comparable to active copers and avoidant copers identified in studies on coping profiles in adolescence by Aldridge and Roesch^31^. Consistent with studies on adults’ coping profiles during the COVID-19 pandemic^7^, these results showed that the majority of children used an active coping style more frequently than an avoidant coping style in response to COVID-19-related stressors. Only a minority of them used avoidant coping styles more or equally frequently compared to the use of active coping styles to deal with stressors during the COVID-19 pandemic.

Our results showed that low and high active copers were related to lower anxiety and depression symptoms than balanced and avoidant copers. Children who relied more on an active coping style than an avoidant coping style had lower anxiety and depression symptoms during the COVID-19 pandemic. Consistent with previous studies, these findings further supported that an active coping style was an adaptive coping style that helped protect children from the negative effects of COVID-19-related stressors, while an avoidant coping style was a maladaptive coping style that might increase the risk of anxiety and depression symptoms during the stressful COVID-19 pandemic^29,41,42^. In studies by Perzow^16^ and Herres^17^, adolescent active copers showed high levels of all coping styles, and they scored the highest on anxiety and depression symptoms. Consistently, our study found that avoidant copers who had the highest level of both avoidant and active coping styles showed the highest anxiety and depression symptoms. High active copers had a larger difference between active and avoidant coping styles than did low active copers. High active copers had lower depression symptoms than low active copers, but they did not differ in anxiety symptoms. Therefore, it was speculated that depression symptoms might be more subject to an increase in the difference between active and avoidant coping styles than anxiety symptoms in the context of the COVID-19 pandemic, where the risk of infection was largely increased.

Balanced copers had more anxiety and depression symptoms than low and high active copers, while they had less anxiety and depression symptoms than avoidant copers. This provided the first evidence that in response to increased stressors in the COVID-19 pandemic (e.g., quarantine and social distancing), having similarly high active and avoidant coping styles was more maladaptive than having a significantly higher level of active coping style but was more adaptive than having a significantly higher level of avoidant coping style. According to reinforcement sensitivity theory^43^, the behavioural inhibition system (BIS) is related to high sensitivity to punishment and motivation to avoid threats, while the behavioural approach system (BAS) is associated with high sensitivity to rewards and motivation to seek rewards^44^. It has been shown that avoidant coping is related to BIS, and active coping is related to BAS^45^. Child balanced copers may have equally activated the BIS and BAS. Therefore, individuals with this profile are motivated by the two incompatible needs to attain potential rewards and avoid threats. The activation of the two inconsistent drives may result in approach-avoidant conflict^46^. When children avoid addressing problems, they may start to feel anxious or depressive about losing the potential rewards; however, when they switch to an active coping style, they feel motivated to avoid problems to reduce stress. Due to this constant conflict between the two motivations, balanced copers might have higher anxiety and depression than children who rely more on an active coping style in response to stressors in the COVID-19 pandemic.

Our study adopted a longitudinal design to investigate how coping profiles prospectively affect changes in anxiety and depression symptoms during the COVID-19 pandemic. Existing longitudinal research on coping profiles is scarce. Such research can provide valuable implications on whether coping profiles affect emotional adjustment to a stressful situation posed by the COVID-19 pandemic over time. Our study showed that overall, anxiety and depression levels decreased from T1 to T2, despite a higher risk of COVID-19 virus infection during T2. Reduction in anxiety and depression might reflect that active–avoidant coping profiles might be effective at reducing anxiety and depression levels in response to COVID-19-related stressors. Our results showed that children’s active–avoidant coping profiles could affect changes in children’s depression levels over a period of 6 months. Although avoidant copers showed the highest level of depression, child avoidant copers reported a larger decrease in depression from T1 to T2 than high active copers and balanced copers during the COVID-19 pandemic, which was counterintuitive. This might suggest that although avoidant coping style contributed to high depression over time, the avoidant-dominant profile was effective at reducing depression levels.

During the COVID-19 pandemic, many children were experiencing COVID-19-related difficulties, including an increased risk of infection and social distancing. Our findings suggest that it might be important to improve children’s coping profiles to help them better cope with the stressors in the COVID-19 pandemic. Focusing on the difference between active and avoidant coping styles is warranted, rather than solely on active or avoidant coping styles. An effective intervention helped children in the COVID-19 pandemic rely more on an active coping style than on an avoidant coping style instead of reducing avoidant coping style alone. Cognitive-behavioural techniques can help prevent anxiety and depression symptoms^47^. In addition to exposure therapy, which helps reduce avoidance, it may be important to apply problem-solving skills training to dispositional avoidant and balanced copers to increase the use of active coping over avoidant coping^48^.

First, we only conducted self-report measures of coping styles, which may be subject to biases. Second, the age range of primary school children was from 9 to 11 years. The limited age range may cause failure to detect the effect of age on psychological variables. Additionally, caution should be taken when generalizing the findings to other age groups. The latent profile analysis revealed a very small group of avoidant copers, which only included 2% of the participants. The unequal sample sizes for each group of active–avoidant coping profiles could affect the type one error level and influence the results. Additionally, the study focused on the distinction between active and avoidant coping styles. Future research could examine whether children’s coping patterns involving other coping styles were related to changes in depression and anxiety during the COVID-19 pandemic. This study found that internalizing symptoms decreased over 6 months during the pandemic. Future studies may investigate whether improving children’s active–avoidant coping profiles is related to changes in internalizing symptoms in the COVID-19 pandemic.

In conclusion, the study revealed four heterogeneous dispositional active–avoidant coping profiles classified based on the difference between active and avoidant coping styles: low active copers, high active copers, balanced copers, and avoidant copers. Our findings suggested that low- and high-active copers were related to fewer anxiety and depression symptoms than balanced and avoidant copers during the COVID-19 pandemic. Additionally, avoidant copers were related to a greater reduction in depression symptoms during the COVID-19 pandemic than high active copers and balanced copers. The findings might provide critical implications for improving children’s coping profiles to reduce internalizing symptoms in children during COVID-19.

## Supplementary Information

Supplementary Information.

## Data Availability

The datasets generated during and/or analyzed during the current study are available from the corresponding author on reasonable request.
