# Supplementary material for: Active and avoidant coping profiles in children and their relationship with anxiety and depression during the COVID-19 pandemic
Source: Sci Rep. 2022 Aug 4;12:13430. doi: 10.1038/s41598-022-15793-4 (PMC9352659; doi:10.1038/s41598-022-15793-4)
Supplement: Supplementary file 1 — Supplementary Information. [file 41598_2022_15793_MOESM1_ESM.docx]

**Supplementary Information**

Title: Active and avoidant coping profiles in children and their relationship with anxiety and depression during the COVID-19 pandemic

**Authors:** Qiaochu, Zhang¹*, Yanlin Zhou^1,2^ , Samuel M.Y. Ho¹

**S.1. Construct validity and internal reliability of the Chinese translation of the Active and Avoidance Coping Scale**

We conducted the confirmatory factor analysis (CFA) to examine the structural validity. For CFA, a non-significant Chi-Square Test of Model fit [40], Goodness of Fit (GFA) > .90 [41], Root Mean Square Residual (RMR) < .08 [42], Comparative Fit Index (CFI) > .90 [43], Tucker Lewis Index (TLI) > .90 [44], and Root Mean Square Error of Approximation (RMSEA) > .80 [42] indicate a good fit of the model.

We first entered the items of the active and avoidant coping scale into the two-factor model based on the original factor structure in the study by Ayers, Sandler [18]. Item 2, item 13, item 11, item 20, item 25, item 8, item 9, item 30, and item 35 had a factor loading below .45. Therefore, these items were deleted from the scale. After deleting items with low factor loadings, the fit indices were acceptable: RMR = .056, GFI = .86, RMSEA = .063, CFI = .89, TLI = .88. Chi-square test of model fit was significant, χ2 (299) = 680.30, p < .001. However, these indexes tend to be significant in a large sample [45]. The final structure of the Chinese version of the active and avoidant coping scale included 26 items (refer to factor loadings for each items below). The internal reliability for the active coping scale was .94, while that for the avoidant coping scale was .74.

**Table S.1 Factor loadings for confirmatory factor analysis of the Chinese translation of the Active and Avoidance Coping Scale for the Chinese children sample (n = 321).**

| **Items** | **Factor 1** | **Factor 2** |
| --- | --- | --- |
| **Factor 1: Avoidant coping** |  |  |
| Item 3 I tried to ignore it. | .668 |  |
| Item 4 I tried to stay away from the problem. | .745 |  |
| Item 17 I tried to put it out of my mind. | .477 |  |
| Item 23 I just forgot about it. | .613 |  |
| Item 32 I avoided it by going to my room. | .555 |  |
| **Factor 2: Active coping** |  |  |
| Item 1 I thought about what I could do before I did something. |  | .645 |
| Item 5 I did something to make things better. |  | .646 |
| Item 6 I told myself that things would get better. |  | .602 |
| Item 7 I reminded myself that I am better off than a lot of other kids. |  | .541 |
| Item 10 I thought about what would happen before I decided what to do. |  | .638 |
| Item 12 I told myself that I could handle this problem. |  | .705 |
| Item 14 I tried to make things better by changing what I did. |  | .702 |
| Item 16 I thought about why it happened. |  | .553 |
| Item 18 I told myself I could handle whatever happens. |  | .651 |
| Item 19 I told myself that in the long run, things would work out for the best. |  | .708 |
| Item 21 I reminded myself that I knew what to do. |  | .718 |
| Item 22 I thought about which things are best to do to handle the problem. |  | .700 |
| Item 24 I told myself that it would work itself out. |  | .630 |
| Item 26 I reminded myself that overall things are pretty good for me. |  | .689 |
| Item 27 I did something to solve the problem. |  | .622 |
| Item 28 I tried to understand it better by thinking more about it. |  | .722 |
| Item 29 I reminded myself about all the things I have going for me. |  | .687 |
| Item 31 I thought about what I needed to know so I could solve the problem. |  | .704 |
| Item 33 I did something in order to get the most I could out of the situation. |  | .548 |
| Item 34 I thought about what I could learn from the problem. |  | .666 |
| Item 36 I tried to figure out why things like this happen. |  | .606 |

**Table S.2 Fit statistics for latent profile analysis models representing one to five coping groups**

| **Number of groups** | **BIC** | **AIC** | **VLMR**  **(p-Value)** | **Adj. LMR (p-Value)** | **BLRT**  **(p-Value)** | **Entropy** |
| --- | --- | --- | --- | --- | --- | --- |
| **1** | 1299.41 | 1284.32 | NA | NA | NA | NA |
| **2** | 1252.59 | 1230.38 | .006 | .008 | 0.00 | 0.89 |
| **3** | 1254.12 | 1216.42 | .05 | .06 | 0.00 | 0.68 |
| **4** | **1207.27** | **1158.24** | **.07** | **.07** | **0.00** | **0.90** |
| **5** | 1218.92 | 1158.58 | .37 | .40 | .36 | 0.75 |

*Note.* The bolded four-group model showed the best model fit.

**Table S.3** **Anxiety and depression symptoms based on the four active-avoidant coping profiles**

|  | 1. Low Active-Dominant | | 2. Coping Balance | | 3. High Active-Dominant | | 4. Avoidant-Dominant | | Difference  Between profiles | Time effect | Profile * Time |
| --- | --- | --- | --- | --- | --- | --- | --- | --- | --- | --- | --- |
|  | M (SD) | | M (SD) | | M (SD) | | M (SD) | |  |  |  |
|  | T1 | T2 | T1 | T2 | T1 | T2 | T1 | T2 | *F* | *F* | *F* |
| Anxiety | 26.95 (21.86) | 12.10  (12.25) | 37.03  (25.78) | 24.03  (20.82) | 22.61  (17.85) | 9.19  (12.09) | 63.44  (21.62) | 35.83  (23.18) | 15.42**  3,1<2<4 | 79.47** | 1.50 |
| Depression | 5.66  (5.93) | 2.10  (3.24) | 7.93  (6.06) | 5.00  (5.24) | 3.79  (4.05) | 1.35  (3.14) | 15.50  (9.35) | 6.67  (7.15) | 18.223**  3<1<2<4 | 64.52** | 4.25* |

** *p* < .001 * *p* < .05.
